# CT-guided cement sacroplasty (CSP) as pain therapy in non-dislocated insufficiency fractures

**DOI:** 10.1007/s00590-017-2001-1

**Published:** 2017-06-26

**Authors:** Reimer Andresen, Sebastian Radmer, Mathias Wollny, Julian Ramin Andresen, Urs Nissen, Hans-Christof Schober

**Affiliations:** 1 Institute of Diagnostic and Interventional Radiology/Neuroradiology, Westkuestenklinikum Heide, Academic Teaching Hospital of the Universities of Kiel, Luebeck and Hamburg, Esmarchstraße 50, 25746 Heide, Germany; 2 Centre for Orthopaedics, Berlin, Germany; 3 Medimbursement, Tarmstedt, Germany; 4 Medical School, Sigmund Freud University, Vienna, Austria; 5 Department of Neurosurgery and Spine Surgery, Westkuestenklinikum Heide, Academic Teaching Hospital of the Universities of Kiel, Luebeck and Hamburg, Heide, Germany; 6 Department of Internal Medicine I, Municipal Hospital Suedstadt Rostock, Academic Teaching Hospital of the University of Rostock, Rostock, Germany

**Keywords:** Insufficiency fracture, Sacrum, Pain therapy, Cement augmentation, Cement leakage, Cement sacroplasty

## Abstract

**Introduction:**

In elderly patients with reduced bone quality, insufficiency fractures of the sacrum are relatively common and are typically associated with severe disabling pain. The objective of the present study was to examine the feasibility of cement augmentation by CSP, to determine post-interventional leakages and other complications, and to present the outcome of pain over the course of 18 months.

**Materials and methods:**

In 23 patients (20 women and 3 men) with an average age of 81.3 (71–92) years, a total of 41 sacral fractures were detected by MRI, 5 of them unilateral and 18 bilateral. Conservative treatment initially performed over a period of 3 weeks did not bring any satisfactory reduction in the severe disabling pain. The indication for intervention was established after an interdisciplinary case conference. The intervention was performed under intubation anaesthesia. Single-shot antibiotic prophylaxis was given routinely immediately prior to the intervention. Under sterile conditions, a Jamshidi needle was then advanced into the respective fracture zone in the sacrum from dorsal to ventral (short axis) or from lateral to medial transiliac (transiliac axis). After removing the inner needle, a flexible osteotome was inserted through the positioned hollow needle and used to extend the spongious space in the fracture zone and thus prepare a cavity for the cement filling. High-viscosity PMMA cement was then inserted discontinuously with the aid of a pressure gauge under low-dose CT control. Cement leakages were determined in the CT image on the day after the intervention, all cement outside of the cortical boundary being rated as a leakage. Pain was documented on a visual analogue scale (VAS) on the day before the intervention, on the second day, and 6, 12, and 18 months after the intervention. Additionally occurring complications were recorded, and the patients were asked to rate their satisfaction after 6 and 18 months.

**Results:**

CSP was technically feasible in all patients. In the control CT scan, sufficient cement distribution and interlocking with vital bone were found along the course of the fracture in the sacrum. An average of 6.0 ± 0.83 ml of cement was inserted per fracture. Leakage was found in 5 of 41 (12.2%) of the fractures treated, although none were symptomatic. The mean pain score on the VAS was 8.8 ± 0.59 before the intervention, a significant pain reduction (*p* < 0.0005) was seen on the second post-operative day, with an average value of 2.1 ± 0.36, and this was stable at 2.2 ± 0.28 after 6, 2.3 ± 0.31 after 12, and 2.2 ± 0.41 after 18 months. Now that they no longer experienced disabling pain, all of the patients were fully remobilised and discharged back home. A high level of patient satisfaction was found after 6 and 18 months.

**Conclusion:**

As a minimally invasive procedure, CSP is an effective treatment method for rapid, significant, and sustained pain reduction.

## Introduction

In elderly patients with reduced bone quality who experience suddenly occurring deep-seated low back pain, the possible presence of an insufficiency fracture in the sacrum should be considered as a differential diagnosis [1, 2]. These fractures usually take a vertical course and are classified into a transalar (type 1), a transforaminal (type 2) and a central zone of the fracture course (type 3) [3, 4]. The fractures can occur unilaterally or bilaterally, whereby the latter may be connected by horizontal branches at the level of the first sacral vertebra. A bilateral type 1 fracture zone is found most commonly in the case of insufficiency fractures [4]. This type of fracture may be accompanied by the most severe disabling deep-seated low back pain, without neurological deficits being seen [2].

Up to now, the standard therapy for sacral insufficiency fractures has been conservative treatment with bed rest and adjuvant analgesic therapy, followed by mobilisation on a walking frame or on forearm crutches with pain-adapted weight-bearing [5]. A problematical aspect of conservative therapy is the increased risk of complications such as deep vein thrombosis, consecutive pulmonary artery embolisms, pneumonia, decubitus ulcers, and depression, while the immobilisation also leads to progressive muscle and bone degeneration [5, 6]. The development of a pseudarthrosis with persistent pain is an additional problem of the conservative approach [6]. In the group of patients with severe disabling pain, the mortality rate under conservative therapy is unacceptably high [7].

As a surgical treatment option, osteosynthesis is available using various different techniques. As a result of the strongly rarefied bone structure, stable conditions cannot always be achieved. The best established method is percutaneous, transiliac screw fixation [8, 9].

As an alternative minimally invasive form of treatment, cement can be injected via hollow needles, analogously to vertebroplasty, this technique first being performed successfully by Garant in 2002 [10]. Rapid and virtually complete pain reduction has been demonstrated with this method, although leakages may occur as a complication and are not always asymptomatic [11, 12].

The objective of this prospective study was an evaluation of the feasibility of CSP with a high-viscosity cement, as well as the post-interventional determination of leakages and further complications, and pain reduction with a follow-up of 18 months.

## Materials and methods

**Fig. 1:**
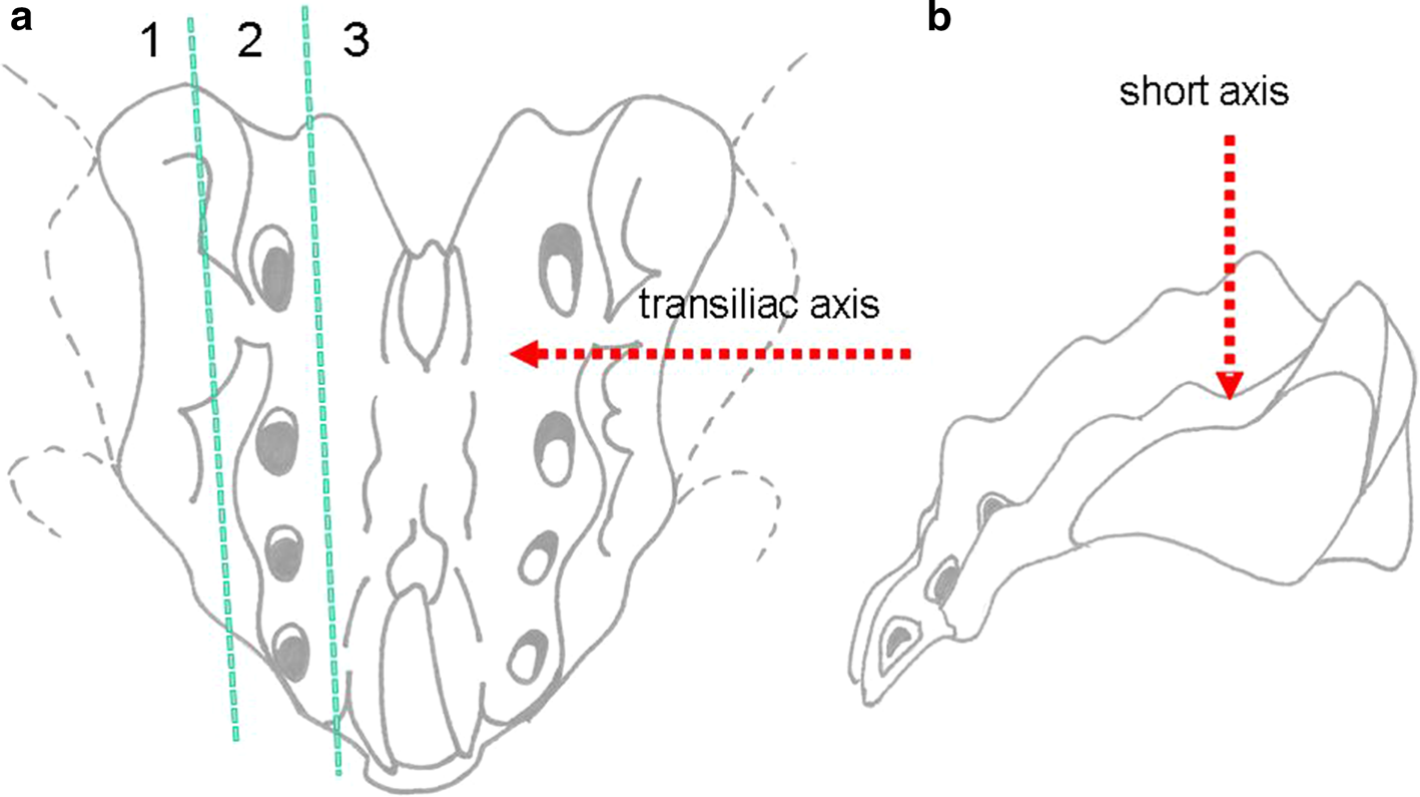
**a**, **b**: Image of the fracture zones in **a** and of the approaches in **b**. All fracture zones according to Denis et al. [3] can be easily accessed with approaches via the so-called short axis and transiliac axis

In 23 patients (20 women and 3 men) with an average age of 81.3 (71–92) years and an average BMI of 25.2 (17.3–31.4) kg/m^2^, a total of 41 isolated sacral fractures, 5 unilateral and 18 bilateral, were detected by MRI. The fractures were classified according to Denis et al. [3] and Rommens and Hofmann [13]. A bone density test was performed on all patients by quantitative computed tomography (QCT). If osteoporosis was present, a supplementary, guideline-compliant pharmacotherapy for osteoporosis [14] was started after healing of the fracture. If a vitamin D deficiency was present, this was immediately corrected. An initially performed conservative treatment with bed rest, pain-adapted mobilisation, and analgetics (non-steroidal anti-inflammatory drugs, morphines) under thrombosis prophylaxis with low molecular weighted heparin over a period of 3 weeks did not achieve a satisfactory reduction in the severe disabling pain. The indication for intervention was established after an interdisciplinary case conference. The intervention was performed under intubation anaesthesia. Single-shot antibiotic prophylaxis was given routinely (cefazolin 2 g i.v.) immediately prior to the intervention. Under sterile conditions, a 10-G Jamshidi needle was then inserted into the respective fracture zone in the sacrum from dorsal to ventral (short axis) or from lateral to medial transiliac (transiliac axis) [4] (Fig. 1). After removing the inner needle, a flexible osteotome was inserted through the positioned working cannula and used to extend the spongious space in the fracture zone and thus prepare a cavity for the cement filling (StabiliT^®^ MX Vertebral Augmentation System—DFINE/Merit Medical). Very-high-viscosity polymethyl methacrylate (PMMA) cement (StabiliT^®^ Bone Cement—DFINE/Merit Medical) was then manually inserted discontinuously under low-dose CT control. Cement leakages were determined in the CT image on the day after the intervention, all cement outside of the cortical boundary being rated as a leakage. Pain was documented on a visual analogue scale (VAS) on the day before the intervention, on the second day, and 6, 12, and 18 months after the intervention.

Statistical analysis of the results was conducted with Prism 5 software (GraphPad). The Wilcoxon test for paired samples (pre-interv. vs. day 2 post-interv., day 2 post-interv. vs. 6 months post-interv., etc.) was used to evaluate the differences between two time points. Statistical significance was characterised as *p < 0.05, **p < 0.005, and ***p < 0.0005.

Additionally occurring complications were recorded, and the patients were asked to rate their satisfaction after 6 and 18 months. Patients with additional fractures in the pelvic girdle were excluded from the study.

## Results

With regard to fracture type, a bilateral Denis 1 was found twelve times (58.6% of the fractures), a bilateral Denis 1–2 six times (29.2% of the fractures), a unilateral Denis 1 three times (7.3% of the fractures), and a unilateral Denis 1–2 fracture zone twice (4.9% of the fractures). A pure Denis 2 or Denis 3 fracture zone was not encountered. All fracture zones extended in length over at least two vertebral body levels. Dislocation of fragments in the area of the fracture zones did not occur. According to Rommens and Hofmann, all fractures were isolated, non-displaced fragility fractures of the sacrum (type II).

In all patients, a severe demineralisation of the axial skeleton could be demonstrated, with bone mineral content values markedly below 50 mg/ml. All patients had a vitamin D deficiency, with vitamin D levels markedly below 30 ng/ml in 18/23 (78.3%) patients and no longer measurable in 5/23 (21.7%) patients.

**Fig. 2:**
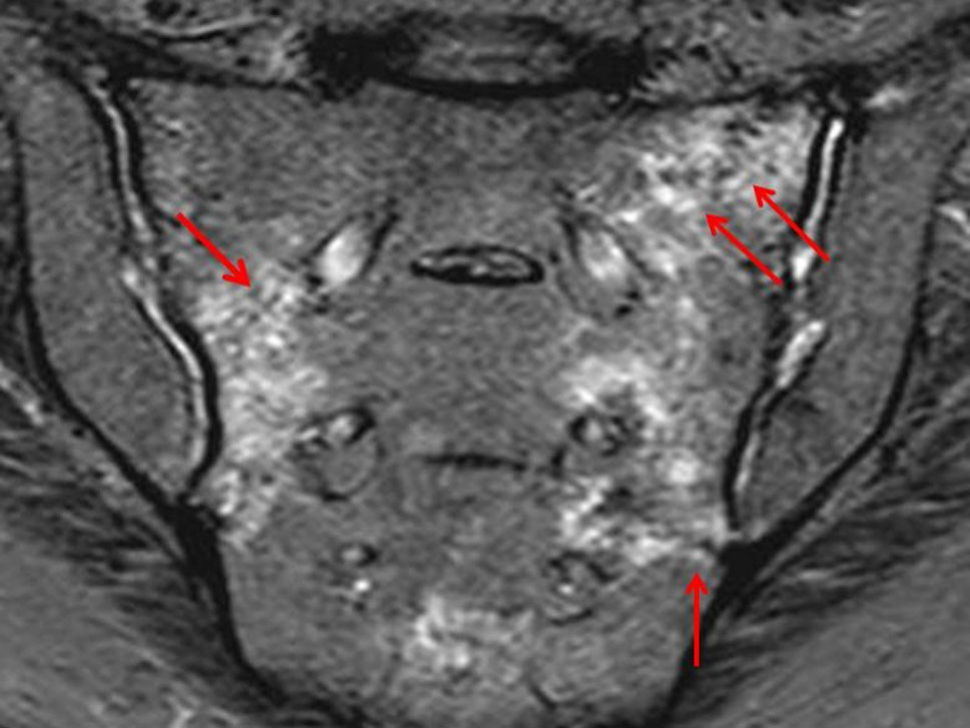
Image of a bilateral sacral fracture, on the right a Denis type 1 fracture zone and on the left with a Denis type 1–2 fracture zone, in the coronal MRI image with strong T2 weighting and partial fat suppression. The bilateral bone marrow oedema is clearly visualised; the* red arrows* mark the fracture lines (colour figure online)

**Fig. 3:**
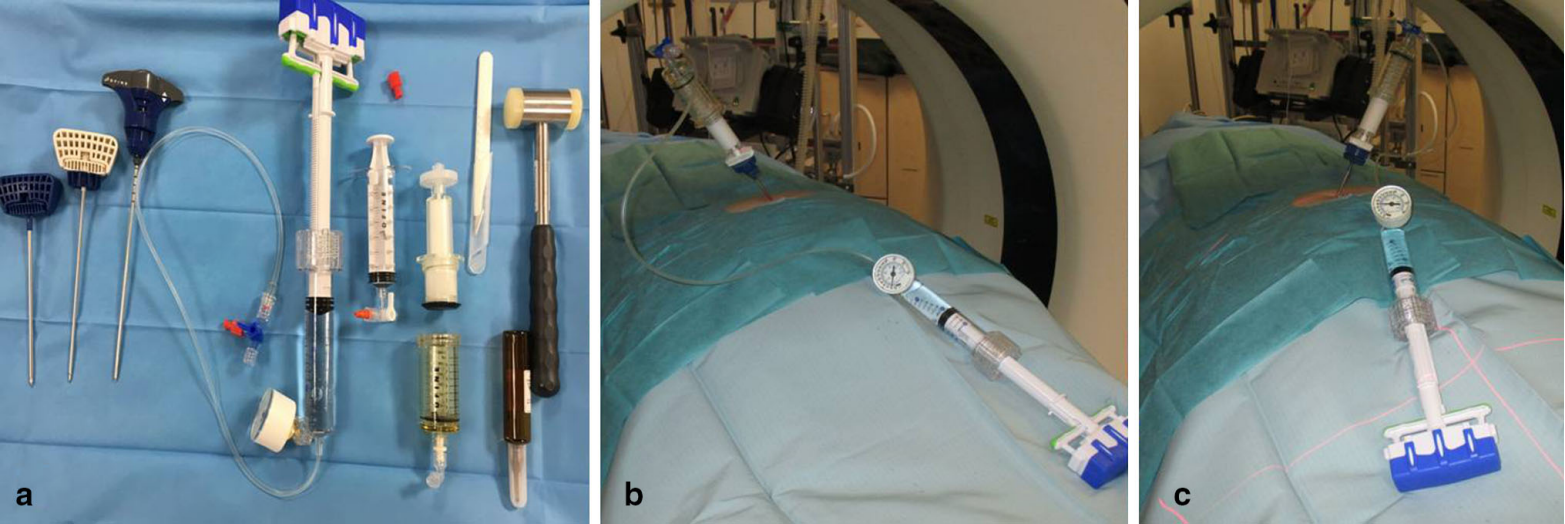
**a**–**c**: In a, instruments of the StabiliT^®^ MX Kit plus sterile hammer. In **b** and **c**, female patient placed in the prone position in the CT with inserted hollow needle and connected cement applicator with pressure gauge. The approach was made via the short axis in each case, in b to treat the* right side*, in a to treat the* left side*

**Fig. 4:**
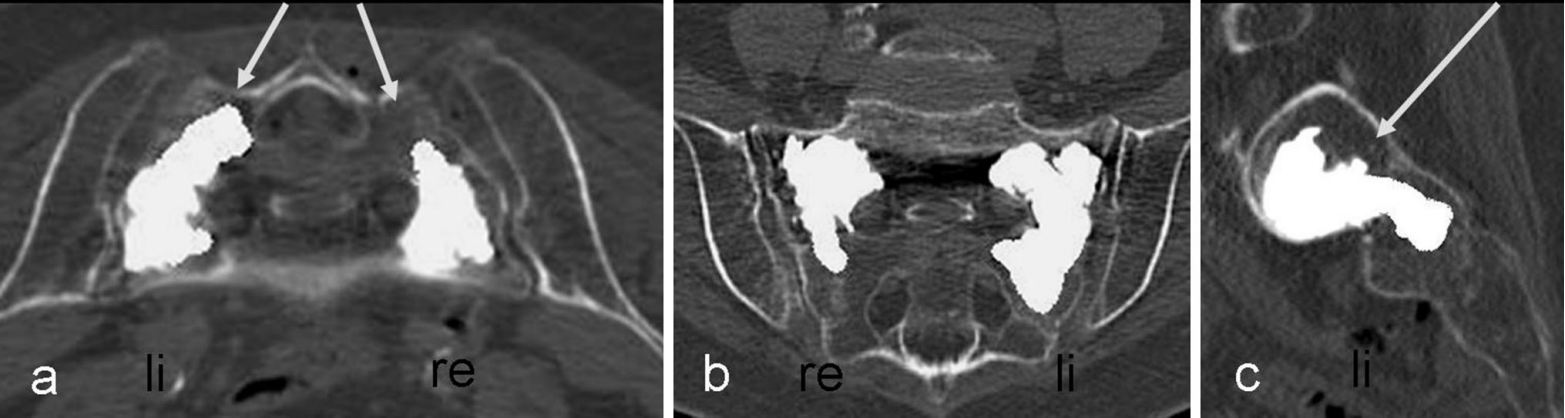
**a**–**c**: Result of the female patient from Fig. 3 **b** and **c** after cement augmentation. In **a**, axial CT image, **b** coronal reformation, and **c** sagittal reformation with a slice thickness of 2 mm. Image of the cement filling located centrally in the fracture zone Denis type 1 on the right and Denis type 1–2 on the* left*. A cement leakage can be ruled out in all three planes. The* arrows* in **a** and **c** mark the approaches via the short axis

CSP was technically fully feasible in all patients. A case example is shown in Figs. 2, 3, and 4.

**Fig. 5:**
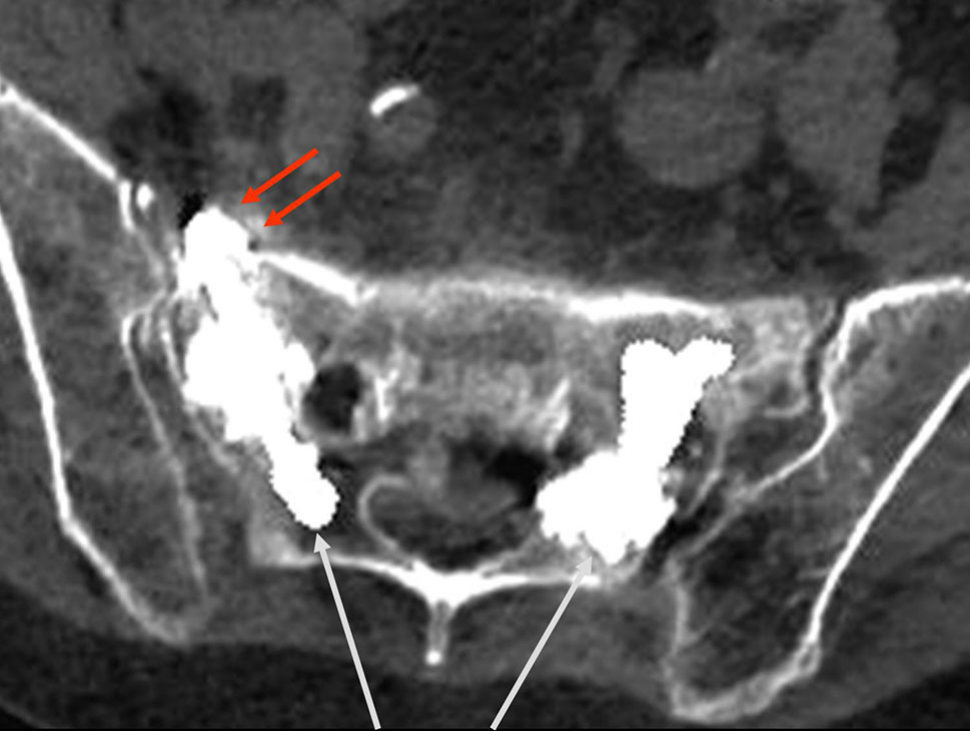
Axial CT image with a layer thickness of 2 mm shows a cement leakage from the fracture zone beyond the cortical boundary of the visceral surface of the sacrum on the* right side* (marked with* red arrows*). On both sides, a Denis type 1 fracture zone was augmented with cement. The approaches via the short axis are marked with* grey arrows* (colour figure online)

In the control CT scans, sufficient cement distribution and interlocking with vital bone were found along the course of the fracture in the sacrum. An average of 6.0 ± 0.83 ml of cement was inserted per fracture. Leakage was found in 5 of 41 (12.2%) of the fractures treated, in 4 of the 5 (80%) leakages cement escaped via a fracture fissure in the direction of the visceral surface of the sacrum (Fig. 5), and in 1 of the 5 (20%) leakages in the direction of the sacroiliac joint. Cement leakage in the direction of the neuroforamina, the intervertebral disc space S1/L5, or the dorsal cortical boundary was not found. None of the leakages were symptomatic.

**Fig. 6:**
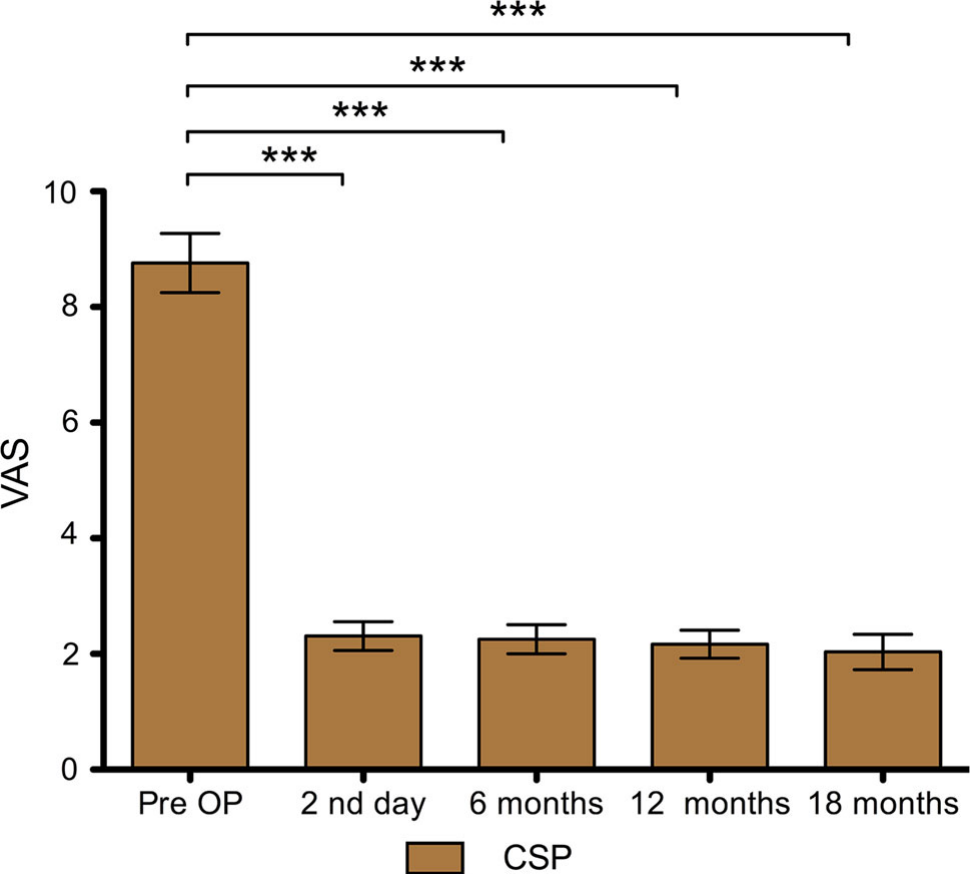
Pain before and over the further course after CSP. Before the intervention, there is a high pain level of on average 8.8 sore points. As early as the second post-operative day, a significant pain reduction to an average of 2.1 score points can be seen. This remains consistently stable after 6, 12, and 18 months. There is no statistically significant difference between the follow-up time points: second post-operative day, 6, 12 and 18 months. The bars show the mean, and the lines represent the standard deviation. Pre-OP, day 2, 6 months (*n* = 23), 12 months (*n* = 22), and 18 months (*n* = 21). *p < 0.05, **p < 0.005, and ***p < 0.0005

The mean pain score on the VAS was 8.8 ± 0.59 before the intervention, a significant pain reduction (*p* < 0.0005) was seen on the second post-operative day, with an average value of 2.1 ± 0.36, and this remained stable at 2.2 ± 0.28 after 6, 2.3 ± 0.31 after 12, and 2.2 ± 0.41 after 18 months (Fig. 6). Now that they no longer experienced disabling pain, all of the patients were fully remobilised and discharged back home or for further rehabilitation on the fourth post-operative day. It was possible to markedly reduce the analgesics and, in some cases, discontinue them completely over the further course. Morphines were no longer needed in any patient.

Post-interventional bleeding or infection was ruled out in all patients. A high level of patient satisfaction was found after 6 and 18 months. Two of 23 (8.7%) patients died over the course, one of pneumonia and another of cancer.

## Discussion

The conservative therapy of sacral insufficiency fractures with immobilisation and pharmacotherapy of pain and osteoporosis lead to an increase in other comorbidities as a result of the immobilisation and often only bring clinical improvement in the long term [5, 7].

Since a rapid analgesic effect with a positive effect on mobility and the activities of everyday life has been repeatedly shown after sacroplasty [7, 10–12, 15–20], this therapeutic option should be considered after an unsuccessful attempt at conservative treatment with persistent disabling pain in the case of non-dislocated sacral fractures according to Rommens and Hofmann [13] type II. In cases of instability or additional fractures of the pelvic girdle, operative treatment is the method of choice [21].

Cement augmentation analogous to vertebroplasty [10–12, 22, 23], balloon kyphoplasty [4, 7, 19], or radiofrequency-targeted vertebral augmentation [18, 20] are possible treatment options. The best clinical experience has been gained with cement injection via a placed hollow needle in accordance with vertebroplasty [10, 22, 23], although cement leakages may occur here, and they are not always asymptomatic [11, 12]. Although they have been described as being safe procedures [20], balloon kyphoplasty [19] and radiofrequency-targeted vertebral augmentation [18] are not always free of leakages either.

The use of a high-viscosity PMMA cement, injected discontinuously with a pressure gauge into a cavity in the fracture zone extended using a flexible osteotome (PowerCURVE—DFINE/Merit Medical), further minimises the risk of cement leakage, although this cannot be prevented completely. Here, the CSP method we used appears to be superior to the conventional vertebroplasty method [12] with regard to the avoidance of cement leakages.

Cement leakages can be further reduced by optimised approaches [4]. Since all fracture zones are fully accessible via the so-called short and transiliac axis, the more complex approach via the so-called long axis [18, 22] does not appear to be necessary.

A good visualisation of the osseous boundaries, the fracture zone, the placed hollow needle as well as the expanding cement filling is imperative to avoid leakages during the intervention. Here, CT-guided cement insertion is markedly superior to fluoroscopy guidance with regard to safety [19, 24].

As a result of the inserted cement filling, an interlocking of the fracture zone and thus a reduction in micro-movements are achieved [25], leading to a reduction in pain [26]. The optimum amount of cement to use has yet to be clarified. Richards et al. conducted biomechanical investigations on 25 cadaveric specimens and showed that stability could be significantly increased after cement augmentation, but that it made no difference whether 3 or 6 ml was injected per side [27]. Since 3 ml of cement is already biomechanically effective for stabilisation [23], the amount of cement to be used should possibly be kept at this volume, because larger cement amounts can increase the risk of leakage. Apart from this, sufficient contact surface of vital bone outside of the inserted cement filling should remain in the fracture zone, so that fracture healing is not completely obstructed here. The limit appears to have been reached with the average amount of 6.0 ml cement that we inserted per fracture. Depending on the extent of the fracture, the approaches, and the different methods used, cement volumes stated in the literature range from 2 to 10 ml [4, 7, 11, 12, 17–23, 26].

## Conclusion

As a minimally invasive procedure, CSP is an effective treatment method for rapid, significant, and sustained pain reduction. On the basis of the published literature, CSP should be given serious consideration in the case of painful non-dislocated isolated insufficiency fractures of the sacrum after conservative therapy has been exhausted and an interdisciplinary case conference held.
